# Relationship between Air Pollution and Urban Forms: Evidence from Prefecture-Level Cities of the Yangtze River Basin

**DOI:** 10.3390/ijerph16183459

**Published:** 2019-09-17

**Authors:** Lijie He, Ying Liu, Peipei He, Hao Zhou

**Affiliations:** 1 Department of Geography and Resource Management, The Chinese University of Hong Kong, Shatin 999077, Hong Kong; 13035128257@163.com; 2 School of Resource and Environmental Science, Wuhan University, Wuhan 430079, China; liuying157@163.com; 3 College of Surveying and Geo-Informatics, North China University of Water Resources and Electric Power, Zhengzhou 450045, China; hepei@ncwu.edu.cn; 4 MOE Key Laboratory of Fundamental Physical Quantities Measurement, School of Physics, Huazhong University of Science and Technology, Wuhan 430074, China

**Keywords:** PM_2.5_, NO_2_, urban form, STIRPAT model, Yangtze River Basin

## Abstract

Urban forms, such as size, shape, density, compactness, and fragmentation, are associated with local air pollution concentrations. However, empirical analyses on how urban form improves or degrades urban air quality are still limited and inconclusive, especially for those rapidly expanding cities in developing countries. In this study, by using the improved STIRPAT (stochastic impacts by regression on population, affluence, and technology) model, the quantitative impact of urban form on near-surface PM_2.5_ and NO_2_ concentrations was identified in the 10 prefecture-level cities of the Yangtze River Basin (YRB) from 2000 to 2013. Trend analyses showed a significant increasing trend in both PM_2.5_ (9.69 × 10^−4^ µg·m^−3^·year^−1^) and NO_2_ (1.73 × 10^−4^ ppb·year^−1^) for the whole study period. Notably, a turning point of PM_2.5_ from increasing to decreasing trends occurred around 2007. In addition, both pollutants showed a spatial agglomeration. The STIRPAT model demonstrated that socioeconomic, transportation and urban form factors played an important role in alleviating the increase of PM_2.5_ and NO_2_. In particular, a 1% decrease in urban extent density (UED) significantly increased NO_2_ by 0.203%, but reduced PM_2.5_ by 0.033%. The proximity index (PI) measured as a city’s compactness was significantly negatively correlated with PM_2.5_ and NO_2_. Conversely, a significant positive relationship of PM_2.5_ and NO_2_ concentrations against the openness index (OI) was observed, an important variable for measuring a city’s fragmentation. In addition, the environmental Kuznets curve (EKC) hypothesis between per capita GDP and PM_2.5_ concentration was confirmed but failed in NO_2_. Overall, this study encouraged a less fragmented and more compact urban form, which helped alleviate local air pollution concentrations by enhancing urban connectivity, reducing vehicle dependence, and facilitating the use of bicycles and walking.

## 1. Introduction

Since the reform and opening up in China, along with the rapid urbanization process, air pollution such as PM_2.5_, PM_10_, NO_2_, SO_2_ and O_3_ has become a serious problem [1,2]. As of 2016, 75.1% of prefecture-level cities in China did not meet the urban air quality standards [3]. As a result, the number of premature deaths caused by air pollution increased from 0.22 million in 2010 to 3.7 million in 2012. In short, poor air quality in China has attracted great attention in recent years.

Air quality is influenced by a variety of meteorological and socioeconomic factors [4,5,6,7,8,9,10]. However, a number of empirical studies, primarily in developed countries, demonstrated that urban form, such as size, shape, population density, compactness, and fragmentation has an important effect on local air pollution [11,12,13,14,15]. For example, Clark et al. [12] reported that changes in population density, centrality and transit supply led to 4%–14% growth in PM_2.5_ concentration in 111 urban zones of United States; impacts equivalent in size to those from meteorological conditions. Likewise, in the United States, fragmented cities experienced low air quality [14]. Moreover, Rodríguez et al. [15] demonstrated that in 249 large urban zones across Europe, a fragmented and highly constructed urban form was associated with higher PM_10_ and NO_2_ concentrations, but densely populated cities experienced higher SO_2_ concentration. One related study was also conducted in East Asia from 2000 to 2010 by Larkin et al. [16], confirming that urban area expansion was strongly correlated with NO_2_ but not PM_2.5_. However, empirical research exploring the relationship between air pollution and urban form was still limited, especially for those rapidly expanding cities in developing countries [17,18,19]. Therefore, in this study, the Yangtze River Basin (YRB) in China, a zone with rapid urban expansion and serious air pollution, was selected as the region of interest.

Even if most of the previous studies suggested that a less fragmented or a more compact urban form was conducive to alleviating local air pollution emissions, the conclusions varied from region to region [11,20,21]. For example, Fan et al. [21] observed that a less fragmented urban form indeed mitigated air pollutant emissions in northern regions of China, but a polycentric form along with the valleys or rivers was encouraged in southern regions of China to improve local air quality. Besides, according to Cho and Choi [22], compact cities might lead to higher population density, thereby increasing local CO and NO_2_ concentrations in Korea. Similarly, Bechle et al. [11] also found that in 83 cities around the world, a city’s compactness was not significantly associated with NO_2_ concentration. Overall, the relationship between air pollution and urban form was inconclusive and required further exploration.

Moreover, most research on this topic has been limited in terms of spatial coverage and temporal continuity [23]. In China, since 2013, 1498 air quality monitoring sites have been launched to provide continuous air pollution observations, including PM_2.5_, PM_10_, NO_2_, SO_2_ and O_3_ [5,24]. However, the data before 2013 was missing and perhaps satellite remote sensing was an ideal technology for addressing this problem. Thus, in this study, long-term annual mean near-surface PM_2.5_ and NO_2_ concentrations were derived from satellite imagery at a high spatial resolution of 0.01° × 0.01°. 

In short, the objectives of this study were to: (1) estimate spatiotemporal trends in near-surface PM_2.5_ (1998–2016) and NO_2_ (1996–2012) concentrations over the YRB, (2) identify spatial autocorrelation in the two air pollutants, if present, and (3) further explore the relationship between air pollution and urban form in the 10 prefecture-level cities of the YRB from 2000 to 2013 by using the improved stochastic impacts by regression on population, affluence, and technology (STIRPAT) model. The organizational structure of this study is as follows. Section 2 introduces urban form data, satellite-based PM_2.5_ and NO_2_ concentrations data, socioeconomic variables, and a series of analytical methods. Section 3 analyzes spatiotemporal trends and spatial autocorrelation in PM_2.5_ and NO_2_ concentrations. The quantitative impact of urban form metrics on the two air pollution concentrations is also estimated in Section 3. Moreover, Section 3 further discusses if there was the environmental Kuznets curve (EKC) hypothesis, an inverted U-shape relationship between per capita income and the two air pollution concentrations [23]. Finally, Section 4 provides a conclusion.

## 2. Data and Methods

### 2.1. Data

#### 2.1.1. Urban Form Data 

**Figure 1:**
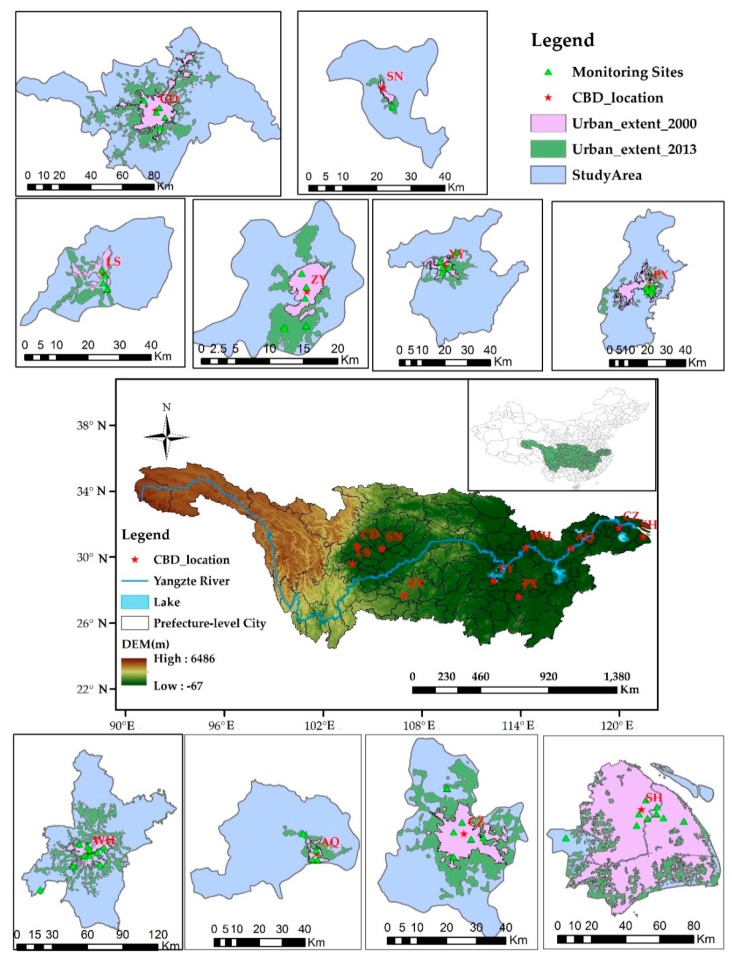
Urban extents of the ten prefecture-level cities over the Yangtze River Basin (YRB) in China from 2000 to 2013. Shanghai (SH), Changzhou (CZ), and Anqing (AQ) are located in the Yangtze River Delta (YRD); Wuhan (WH), Pingxiang (PX) and Yiyang (YY) are located in Central China (CC); the rest of the cities including Chengdu (CD), Zunyi (ZY), Leshan (LS) and Suining (SN) are located all over the Sichuan Basin (SB).

In this study, 10 prefecture-level cities over the YRB with rapid urban sprawl and serious air pollution were selected as the region of interest to reveal the relationship between urban air pollution and urban form. Figure 1 shows, from east to west, Shanghai (SH), Changzhou (CZ), Anqing (AQ), Wuhan (WH), Pingxiang (PX), Yiyang (YY), Zunyi (ZY), Suining (SN), Chengdu (CD) and Leshan (LS). Generally, urban form is defined as the spatial land use configuration of the urban landscape [14,21]. Previous studies monitored the changes of urban form in the process of global urbanization by using various indicators such as urban area, patch number and population density [16]. However, this was not enough and more comprehensive urban form metrics were needed [17]. In this study, considering the size, density, shape, compactness, and fragmentation of a city, five urban form metrics were selected in the 10 prefecture-level cities of the YRB. The relevant vector data were provided by the NYU Urban Expansion Program, UN-Habitat and the Lincoln Institute of Land Policy, and these datasets were freely available online (Table 1).

The detailed description of these urban form metrics and their calculation formula were shown in Table A1. Size in this study referred to the total urban extent area (UEA), an important indicator for measuring urban sprawl, which was calculated from the Landsat 7 (2000) and Landsat 8 (2013) remote sensing images with a pixel resolution of 30 m × 30 m. The density of a given urban extent (UED) was identified as the ratio of its population to the area, in which the population of a given urban extent (UEP) was derived from the census data (2000 and 2013) of China National Census Bureaus. Moreover, fragmentation and compactness represented the shape of a given urban extent, quantified by the openness index (OI) and the proximity index (PI), respectively. Both OI and PI were in the range of 0–1. The OI value close to 1 indicated higher fragmentation [25]. Conversely, higher value of the PI indicated a circular shape of a given urban extent, which was the most compact urban form [25].

#### 2.1.2. Air Pollutant Data

Satellite-retrieved air pollutants provided by the Atmospheric Composition Analysis Group of the National Aeronautics and Space Administration (NASA) were used to identify long-term trends in near-surface PM_2.5_ (1998–2016) and NO_2_ (1996–2012) concentrations over the YRB in this study (Table 1). The annual mean near-surface NO_2_ concentrations at a spatial resolution of 0.01° × 0.01° were derived from GOME, SCIAMACHY and GOME-2 column NO_2_ observations by using a chemical transport model (GEOS-Chem) [26]. Similarly, based on the GEOS-Chem model, the annual mean near-surface PM_2.5_ concentrations (0.01° × 0.01°) were retrieved from MODIS, MISR and SeaWiFS AOD observations, and then they were calibrated to global ground-based PM_2.5_ observations. Previous researches reported a high correlation coefficient (R^2^ = 0.81) between satellite-retrieved PM_2.5_ and global ground-based observation [27]. Such high consistency provided us with enough confidence in using satellite-retrieved air pollutants in this study. However, since there were no valid satellite-retrieved NO_2_ data in 2013, they needed to be calculated from 60 national control sites in the 10 prefecture-level cities of the YRB by using the Kriging interpolation method (Figure 1).

#### 2.1.3. Socioeconomic and Traffic Panel Data

Urban near-surface PM_2.5_ and NO_2_ concentrations were also influenced by several socioeconomic and transportation factors. According to previous research, per capita GDP (PGDP), the proportion of the second industry (PSI), industrial added values (IAV), vehicle ownership (VO) and per capita road area (PRA) were selected as control variables [16,18,19]. These panel data of the ten prefecture-level cities in 2000 and 2013 were obtained from the Statistics Yearbook of China (Table 1).

### 2.2. Methods

**Figure 2:**
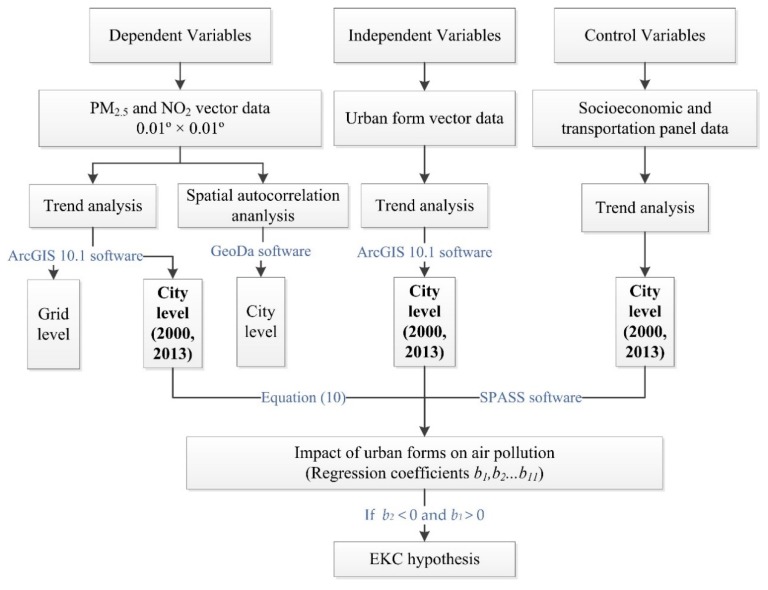
Technical flow chart of this study.

As shown in Figure 2, the technical flow chart in this study was as follows.

Firstly, it was necessary to estimate the long-term trend of PM_2.5_ (1998–2016) and NO_2_ (1996–2012) concentrations at grid level (0.01° × 0.01°) using a linear regression. The regression slope represented the trend. Then, their corresponding statistical significance was further detected by the Mann-Kenddall (MK) method. The statistics S, the variance Var(s)  and the standardized statistics Z were calculated by Equations (1)–(4):

(1) $$S=\overset{n-1}{\underset{i=1}{\sum }}\overset{n}{\underset{j=n+1}{\sum }}sgn\left(X_{j}-X_{i}\right)$$

(2) $$sgn\left(X_{j}-X_{i}\right)=\{\begin{array}{c} +1,\;if\;\left(X_{j}-X_{i}\right)>0\\ 0,\;if\;\left(X_{j}-X_{i}\right)=0\\ -1,\;if\;\left(X_{j}-X_{i}\right)<0 \end{array}$$

(3) $$Var\left(s\right)=\frac{n\left(n-1\right)\left(2n+5\right)-\sum_{p=1}^{q}t_{p}\left(t_{p}-1\right)\left(2t_{p}+5\right)}{18}$$

(4) $$Z=\{\begin{array}{c} \frac{S-1}{\sqrt{Var\left(S\right)}},\;if\;S>0\\ 0,\;if\;S=0\\ \frac{S+1}{\sqrt{Var\left(S\right)}},\;if\;S<0 \end{array}$$

where $X_{j}$ and $X_{i}$ were the near-surface PM_2.5_ and NO_2_ concentrations in the year *j* and *i*, n was the length of the time series, $t_{p}$ was the tied value corresponding to the *p* th number. Only $\left|Z\right|>\left|Z_{\left(1-\alpha /2\right)}\right|$ represented statistical significance in the trend. When the significant level of α = 1%, 5%, and 10%, then their corresponding $\left|Z_{\left(1-\alpha /2\right)}\right|$ were, respectively, 2.58, 1.96 and 1.65. The relevant results were calculated in ArcGIS 10.1 software (Environmental Systems Research Institute, America).

Secondly, in order to investigate the spatial autocorrelation in near-surface PM_2.5_ and NO_2_ concentrations over the YRB, two conventional indexes, namely Global and Local Moran’s I, needed to be calculated in Geoda software (Environmental Systems Research Institute, America). The Global Moran’s I was defined as follows (Equation (5)):

(5) $$I=\;\overset{n}{\underset{i=1}{\sum }}\overset{n}{\underset{j=1}{\sum }}\;\left(x_{i}-\bar{x}\right)\left(x_{j}-\bar{x}\right)/{\left(\overset{n}{\underset{i=1}{\sum }}{\left(x_{i}-\bar{x}\right)}^{2}/n\right)}^{2}\overset{n}{\underset{i=1}{\sum }}\overset{n}{\underset{j=1}{\sum }}\omega_{ij}$$

where $x_{i}$ was the yearly near-surface PM_2.5_ and NO_2_ at the city *i*, *n* was the total number of county-level cities over the YRB, $\omega_{ij}$ was the spatial weight from city *i* to city *j*. The standardized statistics $Z_{I}$ used for detecting the statistical significance of the Global Moran’s I was computed from Equation (6):

(6) $$Z_{I}=\;I-E\left(I\right)/\sqrt{Var\left(I\right)}$$

where E(I)= −1/(n−1), $Var\left(I\right)=E\left(I^{2}\right)-E{\left(I\right)}^{2}$. Generally, the Global Moran’s I ranged from –1 to 1. On the premise of statistical significance, i.e., $Z_{I}$ > 1.65, if Global Moran’s I > 0, then it indicated spatial agglomeration, otherwise, it was spatial dispersion [19]. In terms of the local spatial autocorrelation, LISA (local indicators of spatial association) was selected to spatialize it. LISA is a spatial cluster method based on neighborhood and attributes, dividing the group states into four significant cluster types: high-high, high-low, low-high and low-low [27]. In this study, high-high and low-low patterns represented spatial agglomeration for PM_2.5_ (NO_2_) over the YRB. Conversely, high-low and low-high patterns indicated spatial dispersion. Furthermore, high-high was a hot spot with serious air pollution, while low-low was the cold spot.

After the above two steps, this study undertook an overall analysis of PM_2.5_ and NO_2_ pollution over the YRB in terms of its long-term trend and spatial autocorrelation. However, the reasons for the changes in PM_2.5_ and NO_2_ concentrations required further study. Therefore, this study tried to explore the quantitative impact of urban forms on PM_2.5_ and NO_2_ using a stochastic model (STIRPAT). Moreover, the socioeconomic and transportation metrics were selected as control variables. Notably, the New York City Urban Expansion Plan, UN-Habitat and the Lincoln Land Policy Institute provided relevant urban form indicators only for 2000 and 2013. Therefore, in order to maintain data consistency, all air pollution data, urban form data, socio-economic data and traffic data were selected only for 2000 and 2013 when analyzing the quantitative impact of urban forms on air pollution.

The IPAT model was firstly proposed by Ehrlich and Holdren [28] for revealing the anthropogenic impact on the environment. It was defined as:

(7) I= PAT

where I referred to the anthropogenic environmental impact, P was the population density, A represented the average affluence, generally characterized by GDP, and T was the technology level. According to previous researches [18], the proportion of the second industry (PSI) and the industrial added value (IAV) were selected to represent the technology level. However, the model was inadequate because only a limited number of independent variables were considered. To overcome this weakness, Dietz and Rosa [29] further developed the IPAT into a stochastic model (STIRPAT). It can be expressed as:

(8) $$I_{i}=\alpha \;P_{i}^{b}A_{i}^{c}T_{i}^{d}\varepsilon_{i}$$

After taking logarithms, Equation (8) became as Equation (9):

(9) $$\ln I_{i}=\alpha +b\ln P_{i}+c\ln A_{i}+d\ln T_{i}+\varepsilon_{i}$$

where α was a constant; *i* was the city; b, c, and d were the coefficients of $P_{i}$, $A_{i},$ and $T_{i}$; $\varepsilon_{i}$ was the error term. Additionally, York et al. [30] refined the STIRPAT model by adding quadratic terms of $P_{i}$, $A_{i},$ and $T_{i}$. In this study, according to previous research, the STRIPAT model is improved by adding urban form, socioeconomic and traffic variables. Besides, time dynamic effect (*t*) was also considered in the improved formula:

(10) $$\ln I_{it}=\alpha +b_{1}\ln PGDP_{it}+b_{2}\ln{\left(PGDP_{it}\right)}^{2}+b_{3}\ln SIP_{it}+b_{4}\ln IAV_{it}+b_{5}\ln VO_{it}\;+b_{6}\ln PRA_{it}+b_{7}\ln UEP_{it}+b_{8}\ln UEA_{it}+b_{9}\ln UED_{it}\;+b_{10}\ln OI_{it}+b_{11}\ln PI_{it}+\varepsilon_{i}$$

Actually, the STIRPAT model is composed of a range of multivariate regressions, i.e., Model I, Model II, and Model III. Their corresponding coefficients (b_1_, b_2_ … b_11_) were computed by the 2SLS method. The coefficients (b_1_, b_2_ … b_11_) represented the quantitative impact of the selected socioeconomic, transportation and urban form metrics on PM_2.5_ and NO_2_ concentrations. However, during stepwise regression in SPASS software, these independent variables should have no collinearity with each other, otherwise they would be eliminated. Following the premise, the UEP and UEA independent variables were eliminated from the stepwise regression. 

Finally, according to Equation (10), we could also judge whether there was an EKC hypothesis, an inverted U-shaped relationship between per capita income and air pollutants. This was first proposed by Selden and Song [31] and then empirically examined by a number of researchers [32,33]. The EKC hypothesis states that air pollution increases with income, but then decreases as income increases to a turning point. If $b_{2}$ < 0 and $b_{1}$ > 0 then there was EKC hypothesis, and the turning point was computed by the equation ($-0.5\;b_{2}/b_{1}$).

## 3. Results and Discussions

### 3.1. Near-Surface Air Pollution Concentrations Estimation

#### 3.1.1. Spatiotemporal Trends of Near-Surface PM_2.5_ and NO_2_ Concentrations

**Figure 3:**
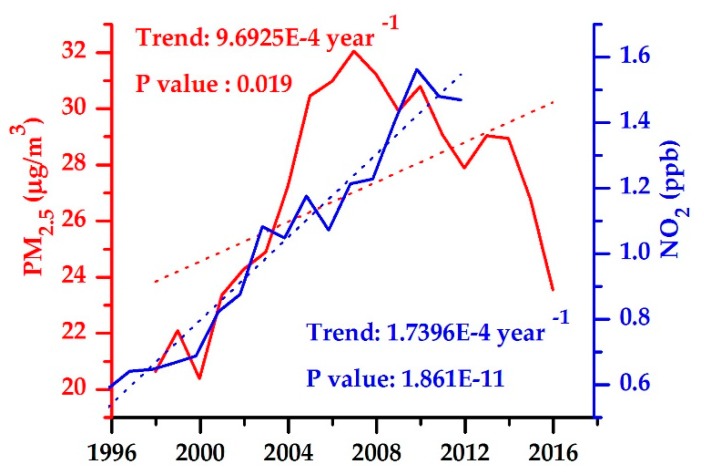
Trend analysis of annual mean near-surface PM_2.5_ (1998–2016) and NO_2_ (1996–2012) over the whole YRB.

**Figure A1:**
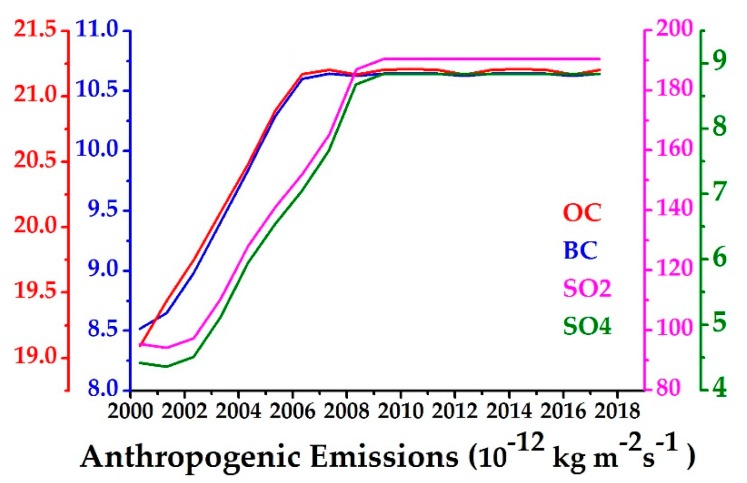
Anthropogenic emissions of organic carbon (OC), black carbon (BC), SO_2_ and SO_4_ derived from the MERRA-2 aerosol reanalysis datasets (https://disc.gsfc.nasa.gov/daac-bin/FTPSubset2.pl?LOOKUPID_List=M2I3NXGAS) over the YRB since 2000.

Figure 3 shows the annual mean trends in near-surface PM_2.5_ (1998–2016) and NO_2_ (1996–2012) concentrations over the whole YRB. Obviously, significant upward trends were observed for both PM_2.5_ (9.69 × 10^−4^ µg·m^−3^·year^−1^) and NO_2_ (1.73 × 10^−4^ ppb·year^−1^) during the entire study period. However, a notable turning point of PM_2.5_ from increasing to decreasing appeared around 2007. The decrease of PM_2.5_ was mainly attributed by the implementation of a series of energy saving and emission reduction policies in China after 2006 [7,8,9,10]. To verify the hypothesis, the main anthropogenic emissions of the YRB including organic carbon (OC), black carbon (BC), SO_2_ and SO_4_ were calculated from the MERRA-2 aerosol reanalysis datasets (Figure A1) from 2000–2017. A good performance of the MERRA-2 product was reported in previous studies [34,35]. As shown in Figure A1, the over-increasing trends of OC, BC, SO_2_ and SO_4_ anthropogenic emissions were curbed efficiently after 2006, and thus decreased near-surface air pollution concentrations. 

**Figure 4:**
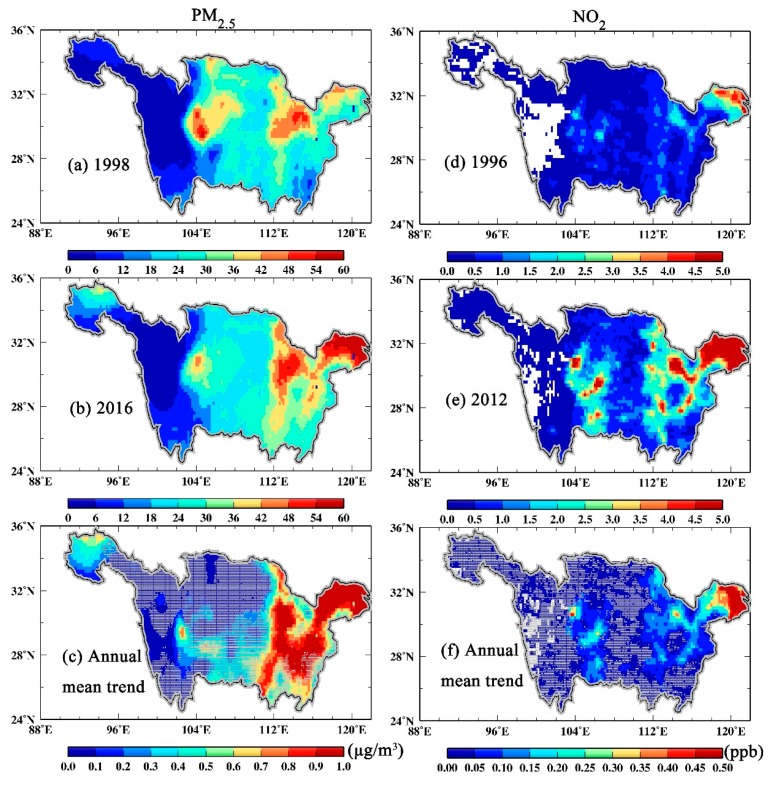
Spatial distributions of annual mean near-surface PM_2.5_ (**c**) and NO_2_ (**f**) concentrations over the YRB. Pixels covered by gray lines refer to those trends not passing the 95% significant level. Besides, annual mean PM_2.5_ (left) and NO_2_ (right) are also shown in 1998 (**a**), 2016 (**b**), 1996 (**d**) and 2012 (**e**), respectively.

Figure 4 depicted the spatial patterns of near-surface PM_2.5_ and NO_2_ trends over the YRB at 0.01° × 0.01° spatial resolution. Also, their corresponding significant levels were detected based on the MK method. The pixels at 95% significant level (*p*-value < 0.05) were marked as no grey line coverage. As illustrated in Figure 4c, significant increasing trends of annual mean near-surface PM_2.5_ (>0.7 µg·m^−3^·year^-1^) appeared over the middle and lower reaches of the YRB. Over these regions, frequent industrialization and urbanization activities always took place, resulting in high annual mean near-surface PM_2.5_ concentrations (>42 µg·m^−3^) from 1998 to 2016 (Figure 4a,b). Notably, another significant increasing PM_2.5_ trend was observed over the source of the YRB, probably caused by the combination of the anthropogenic activities and dust events from Taklimakan Desert [36]. If the increasing trend of PM_2.5_ continues, it will be an alarming condition for the air quality for these regions. In terms of the spatial distribution of NO_2_, high annual mean NO_2_ concentrations (>3.5 ppb·year^−1^) appeared only in the YRD in 1996 (Figure 4c), but expanded to the Central China (CC) and the Sichuan Basin (SB) in 2012 (Figure 4d). Overall, significant increasing trends in annual mean near-surface NO_2_ concentration from 1996 to 2012 were observed over the YRD, CC and SB, of which the largest increase (>0.35 ppb·year^−1^) occurred in the YRD (Figure 4e).

**Figure 5:**
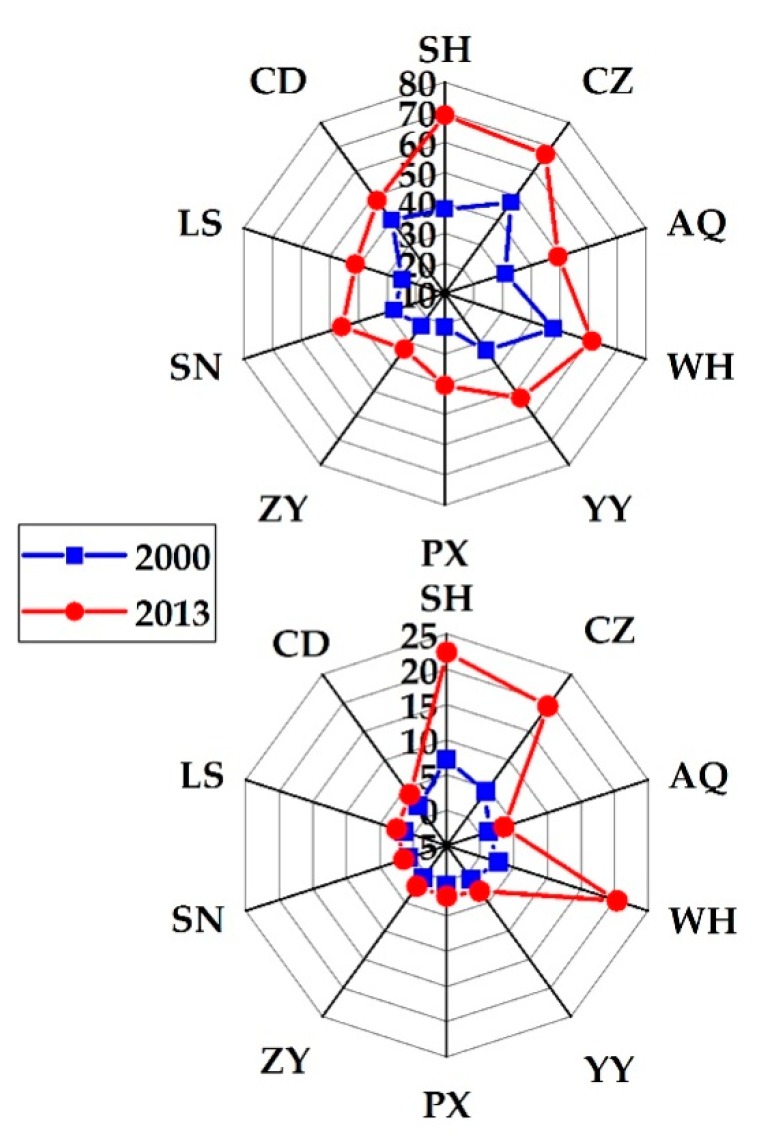
Radar chart of the annual mean near-surface PM_2.5_ (**top**) and NO_2_ (**bottom**) concentrations at the 10 prefecture-level cities of the YRB in 2000 and 2013.

In order to identify the linkage between urban sprawl and urban air pollution, 10 prefecture-level cities were selected from the YRD, CC, and SB regions influenced by serious air pollution. Figure 5 shows the changes in PM_2.5_ (top) and NO_2_ (bottom) concentrations over the 10 urban extents from 2000 to 2013. Overall, the annual mean PM_2.5_ and NO_2_ concentrations in all 10 urban extents in 2013 were higher than those in 2000. The largest increase of PM_2.5_ occurred in SH, with an annual mean growth rate of approximately 4.71%. However, WH experienced the largest increase of the near-surface NO_2_, reaching an annual mean growth rate of approximately 16.82%. According to the ambient air quality standard established by the World Health Organization (WHO), the annual mean near-surface PM_2.5_ concentration should not be exceeded by 10 µg·m^−3^. All of the 10 prefecture-level cities in 2013 were above this threshold. Regarding the near-surface NO_2_ concentration, the WHO recommends not to be exceeded by 20 ppb as the annual mean; 20% of the sample in 2013 was beyond this threshold. These results highlighted that it was necessary to explore which factors derived the degradation of air quality over the YRB.

#### 3.1.2. Spatial Autocorrelation of Near-Surface PM_2.5_ and NO_2_ Concentrations

**Figure 6:**
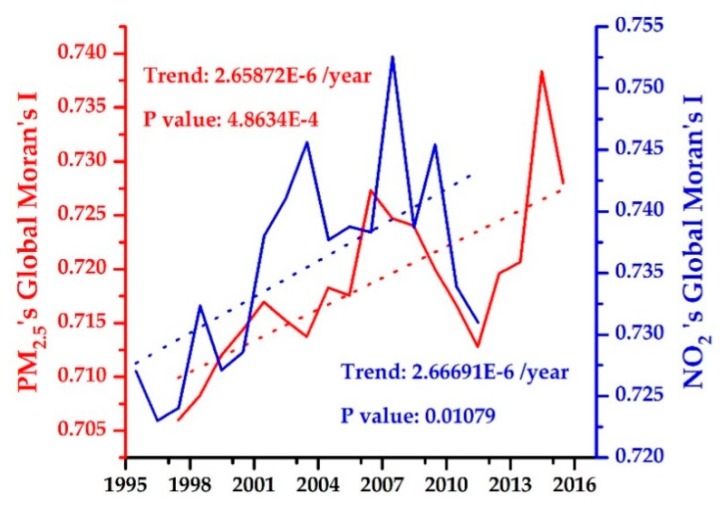
Global Moran’s I variations of the near-surface PM_2.5_ (1998–2016) and NO_2_ (1996–2012) concentrations over the YRB.

Figure 6 showed the Global Moran’s I changes of PM_2.5_ and NO_2_ concentrations over the YRB for the period of 1998–2016 and 1996–2012, respectivley. At 95% significant level, all the Global Moran’s I values were greater than 0, indicating a spatial agglomeration of both PM_2.5_ and NO_2_. In addition, the Global Moran’s I for PM_2.5_ increased from 0.706 in 1998 to 0.728 in 2016, while the Global Global Moran’s I for NO_2_ increased from 0.727 in 1996 to 0.731 in 2012. Result revealed a rapid spatial agglomeration trend in both PM_2.5_ and NO_2_, i.e., the near-surface PM_2.5_ and NO_2_ concentrations over the YRB became more and more clustered during 1998–2016 and 1996–2012, respectively.

**Figure 7:**
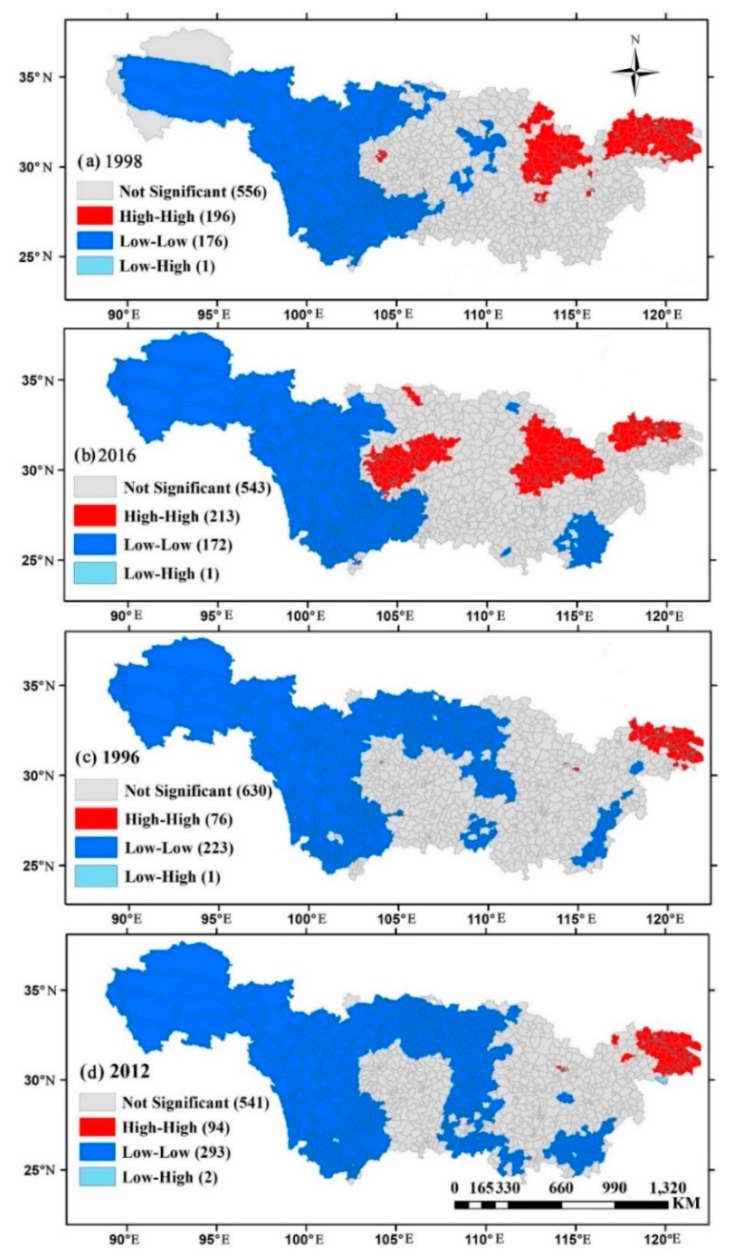
Local indicators of spatial association (LISA) cluster maps of the near-surface PM_2.5_ ((**a**) 1998 and (**b**) 2016) and NO_2_ ((**c**) 1996 and (**d**) 2012) concentrations over the 930 county-level cities of the YRB.

Figure 7 depicts the LISA spatial clustering patterns of near-surface PM_2.5_ and NO_2_ concentrations over the 930 county-level cities of the YRB by using the Local Moran’s I. Generally, there were four LISA cluster patterns: high-high, low-low, high-low and low-high. As shown in Figure 7, for both PM_2.5_ and NO_2_, the high-high and low-low clusters dominated for the whole study period. These results indicated a spatial agglomeration, which was consistent with the Global Moran’s I (Figure 5). Moreover, in terms of PM_2.5_ (Figure 7a,b), no significant changes were observed in the low-low clusters and most of them were located in the upper reaches of the YRB. However, the high-high clusters of PM_2.5_ were mainly located over the YRD and CC in 1998 and then extended to the SB in 2016. Similarly, the high-high clusters of NO_2_ increased from 74 in 1996 to 96 in 2012 (Figure 7c,d). The rapid spatial agglomeration trends of both PM_2.5_ and NO_2_ might be attributed to China’s coordinated regional development policies. A number of heavily polluted enterprises transferred from the east to the west, resulting in the increase of the local air pollutants, and thereby decreased regional inequality [19].

### 3.2. Changes in the Socioeconomic, Transportation and Urban Form Metrics

**Figure 8:**
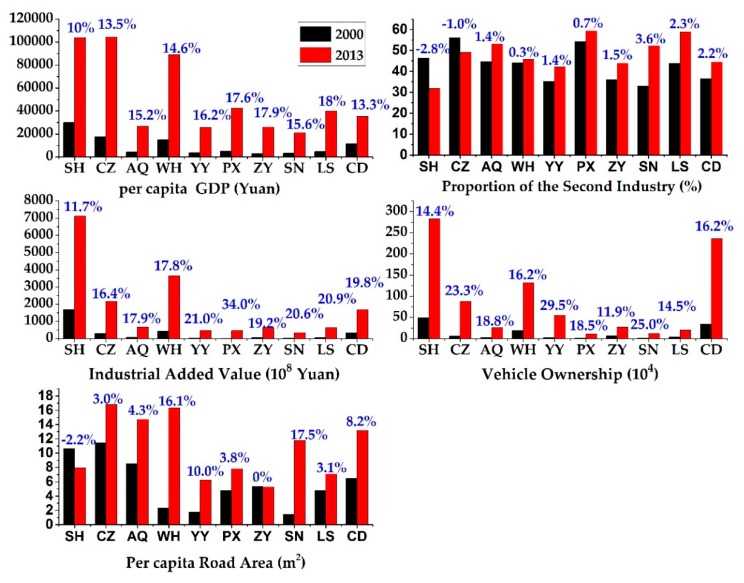
Changes in the socioeconomic factors and transportation metrics at the 10 prefecture-level cities of the YRB during 2000–2013.

Figure 8 shows variations of the socioeconomic factors and transportation metrics. The blue numbers referred to their corresponding annual mean growth rates. Results discovered that the per capita GDP (PGDP) increased significantly at all 10 prefecture-level cities. The largest increase appeared in LS, with an annual mean growth rate of 18%. In terms of the technology level, all 10 prefecture-level cities experienced a significant increasing trend in the industrial added value (IAV). However, only the industrial structures of SH and CZ were optimized, characterized by a decline in the proportion of the second industry (PSI) from 2000 to 2013. Additionally, significant upward trends of vehicle ownership (VO) were also observed in all cities, and thereby increased exhaust gases emission. The per capita road area (PRA) represented a city’s transportation capacity, where higher value of the PRA indicated higher transportation efficiency and less traffic congestion. As illustrated in Figure 8, except for SH, all of the cities experienced a significant increase of the PRA. 

**Figure 9:**
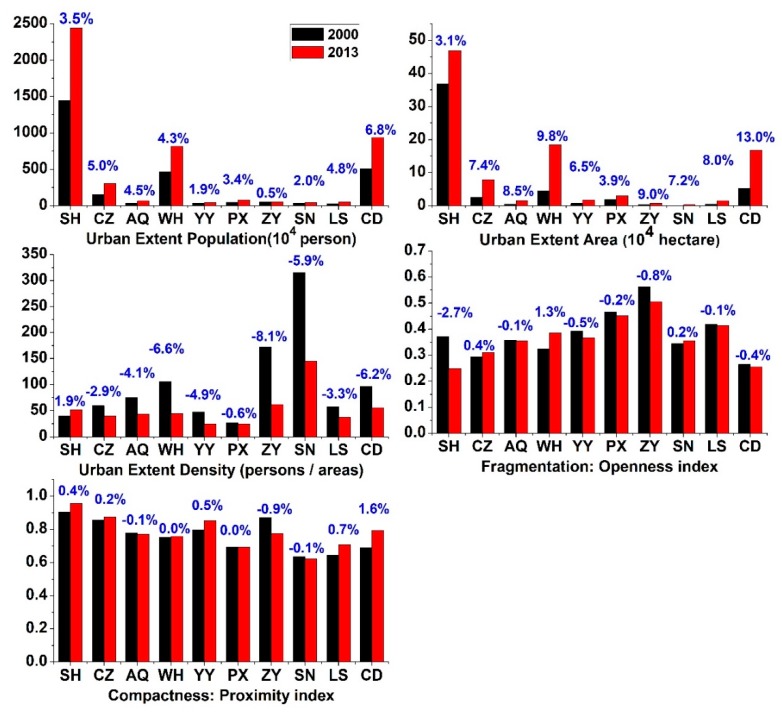
Similar to Figure 8, but for the changes in the five urban form metrics.

Figure 9 depicted dynamic patterns in five urban form metrics. Significant increasing trends were observed in both the urban extent population (UEP) and the urban extent area (UEA). In 2000, the largest UEP appeared in SH (1446 × 10^4^ persons), and increased to 2438 × 10^4^ persons in 2013. By comparison, CD experienced the fastest increase in population between 2000 and 2013, with an annual mean growth rate of 6.8%. Similarly, the fastest expansion of the UEA was also observed in CD, with an annual mean growth rate of 13.0% between 2000 and 2013, followed by WH (9.8%), with urban area increased from 44,273 hectares to 183,723 hectares. However, the smallest growth rate occurred in SH, probably due to the largest urban area in the baseline year (2000) in SH. Overall, results revealed an accelerating process of both population and land urbanizations. 

**Figure A2:**
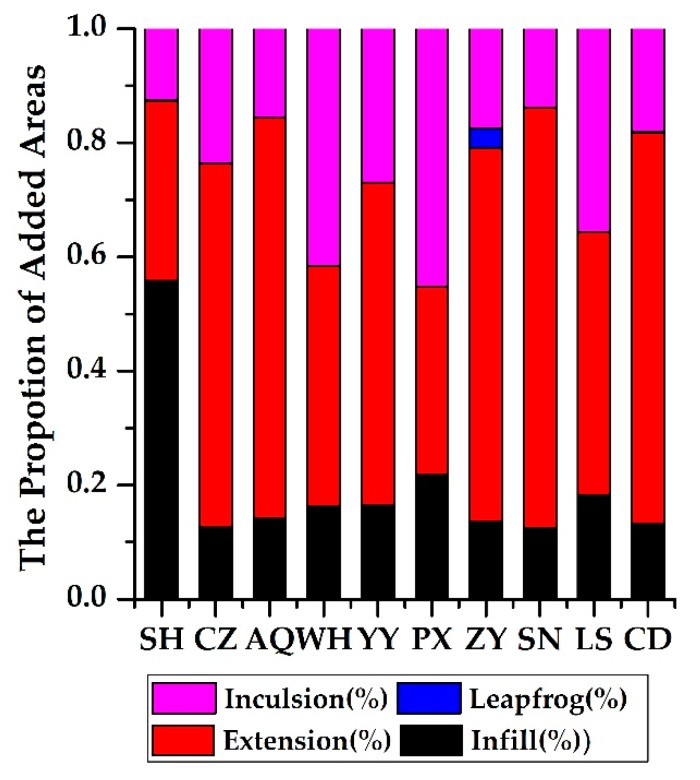
Patterns of the added urban areas at the ten prefecture-level cities of the YRB from 2000 to 2013. Similar to the urban form data, the datasets are also provided by the NYU Urban Expansion Program (http://datatoolkits.lincolninst.edu/subcenters/atlas-urban-expansion/).

However, the urban extent density (UED) of these cities displayed a significant decreasing trend for the whole study period, except for SH. These results indicated that urban land expansion was faster than urban population growth. According to the 2017 China Statistical Yearbook, China has experienced rapid urbanization between 2000 and 2016, in which the land urbanization rate increased by 142.2% but only 72.74% growth was observed in the population urbanization rate. The reason for the phenomenon might be that expansion and leapfrog patterns dominate the land urbanization [14]. In order to verify this statement, the urban sprawl patterns were estimated at the 10 cities across the study period, which were also derived from the NYU Urban Expansion Program (Figure A2). In Figure A2, the expansion type dominated the added areas of all other cities except SH and PX, and thereby reduced the population density of these cities. 

Furthermore, the openness index (OI) was an important indicator for measuring the fragmentation of a given urban extent, ranging from 0 to 1 [25]. Larger OI indicated a more fragmented city, which probably increased the vehicle travelling distance and thus increased the air pollution emissions. Nevertheless, from Figure 9, changes in the OI were different at the 10 cities from 2000 to 2013. CZ, WH, and SN experienced an increasing trend of OI, while a decreasing trend was observed in the other cities. Similarly, uneven changes were observed in the proximity index (PI), a metric for measuring a city’s compactness. It took values in the range of 0–1 [25]. A growing trend in the PI was observed in SH, CZ, YY, LS, and CD, indicating that these cities became more and more compact across the study period.

### 3.3. Drivers of Air Pollutants

Changes in the near-surface PM_2.5_ and NO_2_ concentrations were influenced by a series of socioeconomic factors, transportation indexes as well as urban form metrics [14,15,16,17]. In order to estimate the quantitative impact of these factors on the air pollutants, the improved STIRPAT model was used in this study. 

The Model I was used to identify the quantitative impact of the socioeconomic factors on the near-surface PM_2.5_ and NO_2_ concentrations. Overall, results revealed that the Model I explained 73.9% (adjusted R^2^ = 0.739) and 81.0% (adjusted R^2^ = 0.810) for changes in PM_2.5_ and NO_2_, respectively. The economic growth (PGDP) was positively associated with PM_2.5_ at 99% significant level. In particular, an increase of 1% of PGDP resulted in a 0.554% rise on average in PM_2.5_ concentration. Generally, wealthy cities tend to have denser populations and buildings as well as more vehicles, which may increase air pollution emissions [20]. But on the other hand, it may have cleaner technologies and stricter emission regulations, and thereby contribute to less air pollutant outputs [21]. For example, a negative relationship was observed between PGDP and NO_2_ in this study. Additionally, the proportion of the second industry (PSI) and the industrial added value (IAV) were positively associated with both PM_2.5_ and NO_2_ concentrations. 

Model II extended the basic form of Model I by adding the transportation factors, which were measured as two independent variables of the vehicle ownership (VO) and the per capita road area (PRA). In general, Model II explained 71.4% and 78.5% for changes in the near-surface PM_2.5_ and NO_2_ concentrations, respectively. By comparison, the performance of Model II was lower than Model I. In terms of the VO, it was positively associated with air pollutants at 95% significant level, i.e., a 1% increase in the VO led to a 0.064% and 0.123% increase in PM_2.5_ and NO_2_ concentration, respectively. By contrast, a significant negative relationship (*p*-value < 0.05) was observed between the PRA and air pollutants. The PRA represented a city’s transportation capacity. High PRA values were conducive to reducing traffic congestion, and thus effectively alleviating air pollution emissions, especially the NO_2_ emissions [18]. As shown in Table 2 and Table 3, a 1% increase of the PRA reduced PM_2.5_ and NO_2_ concentrations by 0.072% and 0.087%, respectively.

The Model III could estimate the quantitative impact of the urban form metrics on the near-surface PM_2.5_ and NO_2_ concentrations. Since there was a collinearity between the urban extent population (UEP) and the urban extent area (UEA), the two independent variables were eliminated during the stepwise regression. The rest of the variables, including the urban extent density (UED), proximity index (PI), openness index (OI), were still remained in Model III. In terms of the UED, it was positively associated with PM_2.5_ concentrations but negatively correlated with NO_2_ concentrations at 99% significant level. As discussed in Section 3.2, during 2000–2013, except the SH, all other cities experienced a significant decrease in the UED. The reason was not caused by the decrease of the population, but because land urbanization was faster than urban population growth. The reduction in the UED might lead to a growth in the fragmentation of a given city, thereby increasing the vehicle kilometers of travel and ultimately aggravating transportation-related air pollutant emission, such as NO_2_ emissions. A 1% reduction of UED increased NO_2_ concentrations by 0.203% on average during the study period (Table 3). On the other hand, the lower UED was usually related to a decentralized urban population and construction. On this condition, air pollutants were easier to diffuse and dilute, and thus led to lower pollution concentrations. As shown in Table 2, a 1% decrease of UED results in a 0.033% reduction in PM_2.5_ concentration on average. Recently, Fan et al. [21] also suggested that a high population density could significantly increase SO_2_ emissions but decrease NO_2_ emissions at the 344 prefecture-level cities in China. 

Furthermore, the PI, an important index for measuring the compactness of a given city, was negatively associated with both PM_2.5_ and NO_2_ concentrations. In other words, a compact urban form was conducive to reducing pollution emissions by greater urban connectivity and activity concentration. As illustrated in Table 2 and Table 3, a 1% increase in compactness reduced PM_2.5_ and NO_2_ concentrations by 0.305% and 1.750% on average, respectively. By contrast, a significant positive relationship of the OI against PM_2.5_ and NO_2_ concentrations was observed. These results suggested that the more fragmented urban form tended to increase air pollution emissions by extending vehicle kilometers of travel. Quantitatively, a 1% increase in the fragmentation led to a 0.524% and 1.002% growth, respectively in the PM_2.5_ and NO_2_ concentrations. Similar results were also discussed in cities over the YRD [17], Chinese mainland [18,21], East Asia [16], Europe [15] and United States [14]. All of them suggested that a less-fragmented and more compact urban form was conducive to efficiently mitigating air-pollution emissions. 

**Figure 10:**
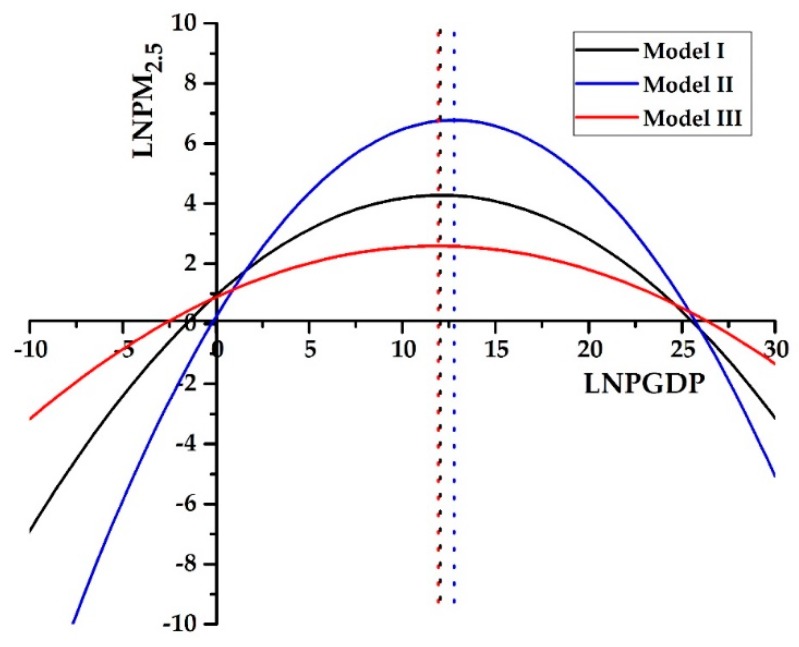
Environmental Kuznets curve (EKC) hypothesis between economic growth and PM_2.5_ concentration in the ten prefecture-level cities of the YRB from 2000 to 2013. The dashed line refers to the turning point of per capita GDP, respectively, in Model I, Model II, and Model III.

Based on Equation (10), Model I, Model II, and Model III could also be used to detect the presence of the EKC between economic growth (PGDP) and air pollution (PM_2.5_ and NO_2_ concentrations). The EKC hypothesis indicated that air pollution concentrations first increase with the growth of the PGDP, but then decrease when the PGDP reaches a turning point. From Table 2 and Table 3, the EKC hypothesis between economic growth and PM_2.5_ concentration was confirmed in all three models but failed in NO_2_ concentration. In addition, Figure 10 showed that only considering the socioeconomic factors (Model I), the turning point of the EKC hypothesis appeared at LNPGDP = 12.0434, i.e., PGDP = 169,987 RMB. Then, by adding the transportation factors (Model II), the turning point was observed in LNPGDP = 12.7875 (PGDP = 357,717 RMB). Finally, through Model III, the turning point happened in LNPGDP = 11.9166 (PGDP = 149,741 RMB). However, the PGDP of all 10 prefecture-level cities of the YRB did not reach the turning point by 2013, in which SH had the highest PGDP of 103,796 RMB. Results suggest that PM2.5 concentration of these cities over the YRB may continue to increase with the PGDP until the turning point is reached. However, the premise is to eliminate the influence of policies and regulations, otherwise it will interfere with the turning point of the EKC hypothesis. For example, as shown in Figure 2, the regional averaged PM_2.5_ concentrations of the YRB initially increased with GDP growth, but then depicted a downward trend after 2007. The turning point was ahead of schedule. The reason might be that the strict pollution control measures were implemented in China after 2007. Besides, the EKC hypothesis might exhibit an N-shaped curve in a long turn [32,33], which needed to be discussed in future studies.

## 4. Conclusions

Based on the improved STIRPAT model, this study tried to investigate the quantitative impact of urban form on the near-surface PM_2.5_ and NO_2_ concentrations in 10 prefecture-level cities of the YRB from 2000 to 2013. Trend analyses revealed that a notable turning point of PM_2.5_ concentrations from increasing to decreasing occurred around 2007. It was probably caused by the implementation of a series of energy-saving and emission-reduction policies in China since 2006. Besides, significant increasing trends in the PM_2.5_ (0.7 µg·m^−3^·year^−1^) and NO_2_ (3.5 ppb·year^−1^) concentrations were observed over the middle and lower reaches of the YRB. Based on the Global Moran’s I, spatial agglomeration was further confirmed in both PM_2.5_ and NO_2_. During the study period, the high-high and low-low clusters dominated the local spatial agglomeration patterns of both air pollutants.

The STIRPAT model further discovered that the socioeconomic and transportation factors were associated with changes in the PM_2.5_ and NO_2_ concentrations. In particular, the growth of the PGDP at the ten prefecture-level cities significantly increased PM_2.5_ concentration but decreased NO_2_ concentration from 2000 to 2013. EKC hypothesis between economic growth and PM_2.5_ concentration was confirmed but failed in NO_2_. The EKC turning point of PM_2.5_ occurred in PGDP = 169,987, 357,717, 149,741 RMB, respectively for Model I, Model II, and Model III. 

Results also highlighted the fact that urban form played an important role in the changes of near-surface PM_2.5_ and NO_2_ concentrations. Since land urbanization was faster than population growth, all cities experienced a decrease in the urban extent density (UED), except for SH. As a result, these cities became more and more fragmented, leading to a significant increase in the transportation-related emissions. For example, a 1% reduction in UED increased NO_2_ concentration by 0.203% on average. Nevertheless, it led to a 0.033% decrease in PM_2.5_ concentration. Additionally, proximity index (PI), an important index measured as a city’s compactness, was significantly negatively associated with PM_2.5_ and NO_2_ concentrations. A 1% increase in PI led to a significant decrease of 0.305% (1.705%) in PM_2.5_ (NO_2_) concentration. On the contrary, there was a significant positive relationship of PM_2.5_ and NO_2_ concentrations against openness index (OI), an indicator used for measuring a city’s fragmentation. Between 2000 and 2013, a 1% increase in OI would result in a growth of 0.524% (1.002%) in PM_2.5_ (NO_2_) concentration. 

Overall, this study might provide a better understanding of the relationship between urban form and air quality from an empirical analysis. It suggested that a more compact and a less-fragmented urban form was conducive to alleviating air pollution emissions at prefecture level by enhancing urban connectivity and reducing vehicle dependence. However, several limitations need to be addressed in future research. For example, high-resolution (e.g., 250 m) PM_2.5_ estimations were still unable to be related with the urban landscape or small geographical units, which is crucial for analyzing the urban pollution structure. The new AOD data with a 160 m spatial resolution retrieved by the Gaofen-1 (GF) may be an ideal technology for addressing this problem [37].

## Figures and Tables

**Table 1:** Data sources and descriptions.

Data	Spatial Resolution	Temporal Coverage	Data Type	Data Source
Urban form data	Prefecture-level city	2000, 2013	Vector data	(http://datatoolkits.lincolninst.edu/subcenters/atlas-urban-expansion/)
Near-surface PM_2.5_	0.01° × 0.01°	1998–2016	Raster data	Atmospheric Composition Analysis Group (http://fizz.phys.dal.ca/~atmos/martin/?page_id=140)
Near-surface NO_2_	0.01° × 0.01°	1996–2012	Raster data	Atmospheric Composition Analysis Group (http://fizz.phys.dal.ca/~atmos/martin/?page_id=140)
	Prefecture-level city	2013	Ground data	China Air Quality Real-time Monitoring platform (http://113.108.142.147:20035/emcpublish/)
Socio-economic and Traffic data	Prefecture-level city	2000, 2013	Panel data	Statistics Yearbook of China (http://tongji.cnki.net/kns55/Navi/NaviDefault.aspx)

**Table 2:** Multivariate relationship of socioeconomic, transportation and urban form metrics with the near-surface PM2.5 concentrations calculated by the improved stochastic impacts by regression on population, affluence, and technology (STIRPAT) model. The standard deviation is shown in parentheses. PGDP refers to per capita GDP; PSI refers to the proportion of the second industry; IAV refers to industrial added values; VO refers to vehicle ownership; PRA refers to per capita road area; UED refers to the density of a given urban extent; OI refers to openness index; PI refers to proximity index. * *p*-value < 0.1, ** *p*-value < 0.05, *** *p*-value < 0.01.

Explanatory Variables	Model I	Model II	Model III
*Socioeconomic factors*			
LNPGDP	0.554 (0.858) ***	1.023 (1.094) **	0.286 (1.074) ***
LN(PGDP)^2^	−0.023 (0.041) ***	−0.040 (0.051) **	−0.012 (0.050) **
LNPSI	0.316 (0.348) **	0.625 (0.574) *	0.006 (0.576) *
LNIAV	0.048 (0.084) *	0.068 (0.109) *	0.029 (0.085) *
*Transportation factors*			
LNVO		0.064 (0.122) **	0.023 (0.114) *
LNPRA		−0.072 (0.087) **	−0.029 (0.085) **
*Urban form factors*			
LNUED			0.033 (0.098) **
LNPI			−0.305 (0.453) **
LNOI			0.524 (0.216) **
Constant	0.947 (3.440) ***	0.239 (3.746) **	0.884 (3.943) **
Adjusted R^2^	0.739	0.714	0.773

**Table 3:** Similar to Table 2, but for the near-surface NO_2_ concentrations.

Explanatory Variables	Model I	Model II	Model III
*Socioeconomic factors*			
LNPGDP	−1.058 (1.875) **	−0.330 (2.430) **	−0.943 (1.732) **
LN(PGDP)^2^	0.062 (0.089) **	0.029 (0.114) **	0.053 (0.081) **
LNPSI	0.209 (0.760) **	−0.297 (1.275)	0.435 (0.928) *
LNIAV	0.366 (0.183) **	0.366 (0.242) **	0.400 (0.196) **
*Transportation factors*			
LNVO		0.123 (0.271) **	0.016 (0.184) **
LNPRA		−0.087 (0.192) **	−0.150 (0.137) **
*Urban form factors*			
LNUED			−0.203 (0.157) ***
LNPI			−1.750 (0.731) **
LNOI			1.002 (0.348) **
Constant	2.280 (7.523) **	1.103 (8.324) *	3.057 (6.359) **
Adjusted R^2^	0.810	0.785	0.953

* *p*-value < 0.1, ** *p*-value < 0.05, *** *p*-value < 0.01.

## Appendix A

**Table A1:** Descriptions of urban form metrics used in this study.

Index	Equation	Description	Significance
Urban extent population (UEP)	$\mathrm{UEP}=\;\overset{n}{\underset{j=1}{\sum }}a_{i}$	a*_j_* = total population of urban patch *j*. n = number of urban patches.	Measures the size of a given urban extent.
Urban extent Area (UEA)	$\mathrm{UEA}=\overset{n}{\underset{j=1}{\sum }}b_{j}$	b*_j_* = total area (hectare) of urban patch *j*.	
Urban extent density (UED)	UED = UEP/UEA	The ratio of UEP to UEA.	Measure of urban sprawl. Increase in the UED results in growth of compactness.
Openness index (OI)	\	The average share of open space pixels within the Walking Distance Circle (a circle with an area of 1 km^2^ and a radius of 564 meters) of each build-up pixel.	Measure of the fragmentation of a given urban extent. The value ranges from 0 to 1. Increase in OI results in a higher fragmentation.
Proximity index (PI)	PI = L/L’	L = the average beeline distance of all points in the equal area circle to city hall. L’ = the average beeline distance of all points in the urban extent to city hall.	Measure of the compactness of a given urban extent. The value ranges from 0 to 1. The closer the PI is to 1, the more compact the city will be.
